# Role play of regulatory T cells and resolvins in type 2 diabetes

**DOI:** 10.3389/fimmu.2026.1748000

**Published:** 2026-03-23

**Authors:** Sandhya Krishnan, Shriraam Mahadevan, Hanuman Prasad Sharma, Undurti Narasimha Das, Kaviarasan Kuppan

**Affiliations:** 1 Department of Biomedical Sciences, Faculty of Biomedical Sciences and Technology, Sri Ramachandra Institute of Higher Education and Research, Chennai, India; 2 Department of Endocrinology, Sri Ramachandra Medical College, Chennai, India; 3 Scientist – II Bioanalytics Facility, Centralized Core Research Facility, All India Institute of Medical Sciences (AIIMS), New Delhi, India; 4 UND Life Sciences, Battle Ground, WA, United States

**Keywords:** diabetes, insulin resistance, lipid mediators, obesity, resolvins, T regulatory cells (Tregs)

## Abstract

Low-grade systemic inflammation is seen in type 2 diabetes mellitus (T2DM), which is also considered the underlying mechanism for the occurrence of major complications observed in these patients. In developing countries like India, prediabetes/insulin resistance is often overlooked. Specialized pro-resolving mediators (SPMs) are a family of anti-inflammatory lipid molecules that are needed to resolve inflammation to maintain immune balance and prevent complications due to DM. Treg cells are a subset of CD4^+^ T cells that express IL-2 receptor alpha chain CD25 and FOXP3 transcription factor to maintain immune tolerance during diabetes. In this context, the role of resolvins in the regulatory function of Treg cells assumes significance in the prevention and management of diabetic complications. In the current review, we explore the functions of Tregs and resolvins and their potential implications in managing insulin resistance, obesity, and type 2 diabetes.

## 1 Introduction

Diabetes, characterized by hyperglycemia, is a chronic metabolic disorder prevalent globally with higher morbidity and mortality rates (1). The quality of life in diabetic patients is compromised due to the complications that result from dysglycemia involving many target tissues (2). The incidence of diabetes mellitus in India is high, affecting approximately 74 million by the year 2021, and is expected to increase to 700 million by 2045. It is predicted that undiagnosed diabetics in India are approximately 57%, constituting approximately 43 million people in the year 2019, and it is estimated to be approximately 548 million by the year 2045, including those who have impaired glucose tolerance (IGT) or the prediabetes stage (3). An increase in insulin resistance, especially hepatic insulin resistance that results in impaired fasting glucose and eventually leads to the development of T2DM, is common in the Indian population (4–7). Other systemic factors, like variability in blood pressure, heart rate, and lipid parameters (dyslipidemia), also contribute to the aggravation of diabetic complications like nephropathy and diabetes-induced cardiovascular disorders (8). Persistent inflammation in diabetes seems to account for the development of insulin resistance (9). Elevated levels of pro-inflammatory cytokines IL-1β, IL-6, TNF-α, and acute-phase proteins like CRP observed in obesity suggest that the activation of immune cells is common in T2DM (9, 10). In diabetic patients and mouse models of diabetes, pathogen recognition is known to be impaired along with dysfunctional leukocyte, monocyte, neutrophil, and natural killer cell recruitment and suppression of appropriate cytokine production (11). This suggests that inappropriate immune regulation plays a major role in the pathogenesis of diabetic complications. In this context, the role of Tregs in the regulation of immune response is important.

### 1.1 Tregs

Tregs are a subset of CD4^+^ T cells, expressing IL-2 receptor alpha chain CD25 and FOXP3 transcription factor. They are mostly thymic-derived, although a few mature in the peripheral lymphoid organs, which are called induced Tregs (iTregs), while a set of endogenously induced Tregs (pTregs) are produced because of antigen stimulation (12). Markers for Tregs include CD25, cytotoxic T lymphocyte-associated antigen 4 (CTLA-4), glucocorticoid-induced tumor necrosis factor receptor family-related gene (GITR), lymphocyte activation gene-3 (LAG-3), CD127, and Forkhead/winged-helix transcription factor box P3 (FOXP3). Of all the markers described, FOXP3 is the most reliable. Tregs are involved in maintaining immunologic self-tolerance and immune balance and preventing autoimmune diseases, in addition to their crucial functions, such as suppression of pathogen-induced immunopathology and suppression of T-cell activation in response to weak stimuli (13). Tregs display different phenotypes and functions based on their tissue sites due to their high plasticity and unstable nature. Colon tissue Tregs, skeletal muscle Tregs, and oral mucosa Tregs are shown to inhibit inflammation (14). Even though inflammation is the key to many diseases, especially in the pathobiology of T2DM, it is noteworthy that it is a complex process that needs close attention.

## 2 SPM and inflammation

Inflammation essentially involves two phases: the initiation phase and the resolution phase. The initiation phase involves the four cardinal signs (redness, heat, swelling, and pain—rubor, calor, tumor, and dolor, respectively) that are associated with the production of chemical messenger including but not limited to chemokines, cytokines, prostaglandins (PGs), leukotrienes (LTs), and thromboxanes (TXs) (collectively called eicosanoids). PGs, LTs, and TXs are pro-inflammatory in nature and regulate the leukocyte trafficking to the site of inflammation (15). The resolution phase involves the clearance of microbes, polymorphonuclear neutrophils (PMNs), and other debris, clearing the infection (16). Studies have revealed that both the phases of inflammation are regulated by lipids derived from arachidonic acid (AA; 20:4 *n*−6) and other polyunsaturated fatty acids (PUFAs) with very specific and target-oriented changes in the concentrations of pro- and anti-inflammatory lipids (mainly eicosanoids). The initial inflammatory phase is characterized and regulated by the production of pro-inflammatory eicosanoids, especially PGs, LTs, and TXs. The resolution phase is dominated by a lipid switch leading to the production of specialized pro-resolving mediators (SPMs), which include lipoxins, resolvins, protectins, and maresins derived from AA, eicosapentaenoic acid (EPA; 20:5 *n*−3), and docosahexaenoic acid (DHA; 22:6 *n*−3), which clear neutrophils from the site of inflammation, facilitate macrophage efferocytosis, and reprogram the inflammatory events that results in the abrogation of immune cell infiltration and initiates tissue repair mechanisms (17). Endogenous SPM production is observed in body fluids (like Cerebrospinal Fluid (CSF), urine, and peripheral blood), organs (like spleen and lymph node), and others like pro-resolving macrophages and apoptotic neutrophils (18).

SPMs are a family of pro-resolving molecules produced by an enzymatic process from essential fatty acids (EFAs), mainly from PUFAs. They are classified further into four different classes—lipoxins, resolvins, protectins (PRTs), and maresins (MaRs). It is likely that each class of these molecules plays a distinct and specific role in the inflammation resolution process, hence the name “specialized pro-resolving mediators” (SPMs). The precursor for LXs is AA; RSVs are derived from EPA and DHA, with the E series derived from EPA and the D series from DHA; PRTs and MaRs are synthesized from DHA.

**Figure 1:**
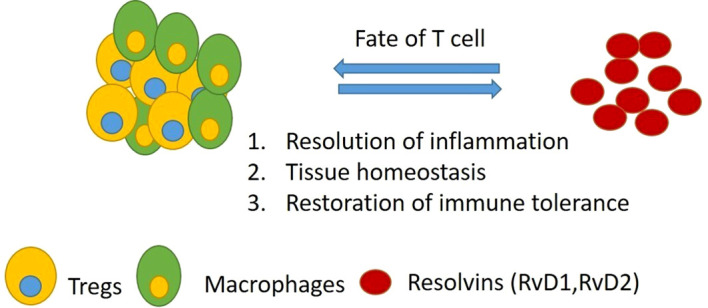
Tregs and Resolvins Link (SPM): SPMs and pro-inflammatory lipids promote or inhibit, in opposite ways, which exhibit pro- or anti-inflammatory actions based on the local environment of SPM secretion; this, in turn, can also influence T-cell fate. SPM derived from Tregs and macrophages exhibits anti-inflammatory functions, maintaining homeostasis. Created using the BioRender software. SPM, specialized pro-resolving mediator.

PGs, LTs, and TXs derived from AA are the primary mediators of the initial phase of inflammation that aid in recruiting neutrophils and regulate blood flow and vascular permeability (16). The resolving phase of inflammation is regulated by SPMs and regulatory T cells that also seem to have an important role in the pathobiology of inflammatory events observed in obesity, T2DM, and the molecular pathology of diabetic complications. This review specifically focuses on the link between SPMs, primarily resolvins, and T_reg_ cells’ contribution to the inflammatory process of insulin resistance and T2DM (**Figure 1**).

### 2.1 Resolvins

The synthesis of resolvins involves the conversion of EPA and DHA into various oxygenase products. The generation of resolvins is complex and involves multiple enzyme-catalyzed reactions. EPA is converted to the E series of resolvins by the action of 5- LOX and subsequent enzymatic reactions. DHA is converted to D-series resolvins by the action of 15- LOX and subsequent enzymatic reactions. Resolvins are crucial to suppress inappropriate inflammation and tissue repair (19). E-series resolvins (RvE1 to RvE3) are derived from either acetylated COX-2 or cytochrome P450 monooxygenases, which leads to the formation of intermediate unstable hydroperoxy compounds (18-HEPE), which are further converted to RvE1 or RvE2 by 5-lipoxygenase (5- LOX) and RvE3 by 15-lipoxygenase (15- LOX). The acetylated COX-2 predominantly produces 18R-HpEPE rather than 18S-HpEPE, leading to the 18R-E-series called the aspirin-triggered E-series resolvins. D-series resolvins are produced from DHA by the enzymatic action of COX-2, leading to the formation of intermediate product 17-hydroxydocosahexaenoic acid (17-HDHA), which is converted to RvD1–RvD6 upon the enzymatic action of 5- LOX. These resolvins differ in their stereochemistry at the 17th carbon position (20, 21).

## 3 Tregs in obesity

Obesity and insulin resistance are closely interconnected and are associated with low-grade systemic inflammation, oxidative stress, endoplasmic reticulum stress, and dysfunction in adipocyte status (22–24). FOXP3, PPAR-*γ*, *Id2*, IL-33, IL-10, and IL-35 are some of the major regulators of adipose tissue that trigger low-grade inflammation, which can ultimately lead to the development of T2DM (13).

**Figure 2:**
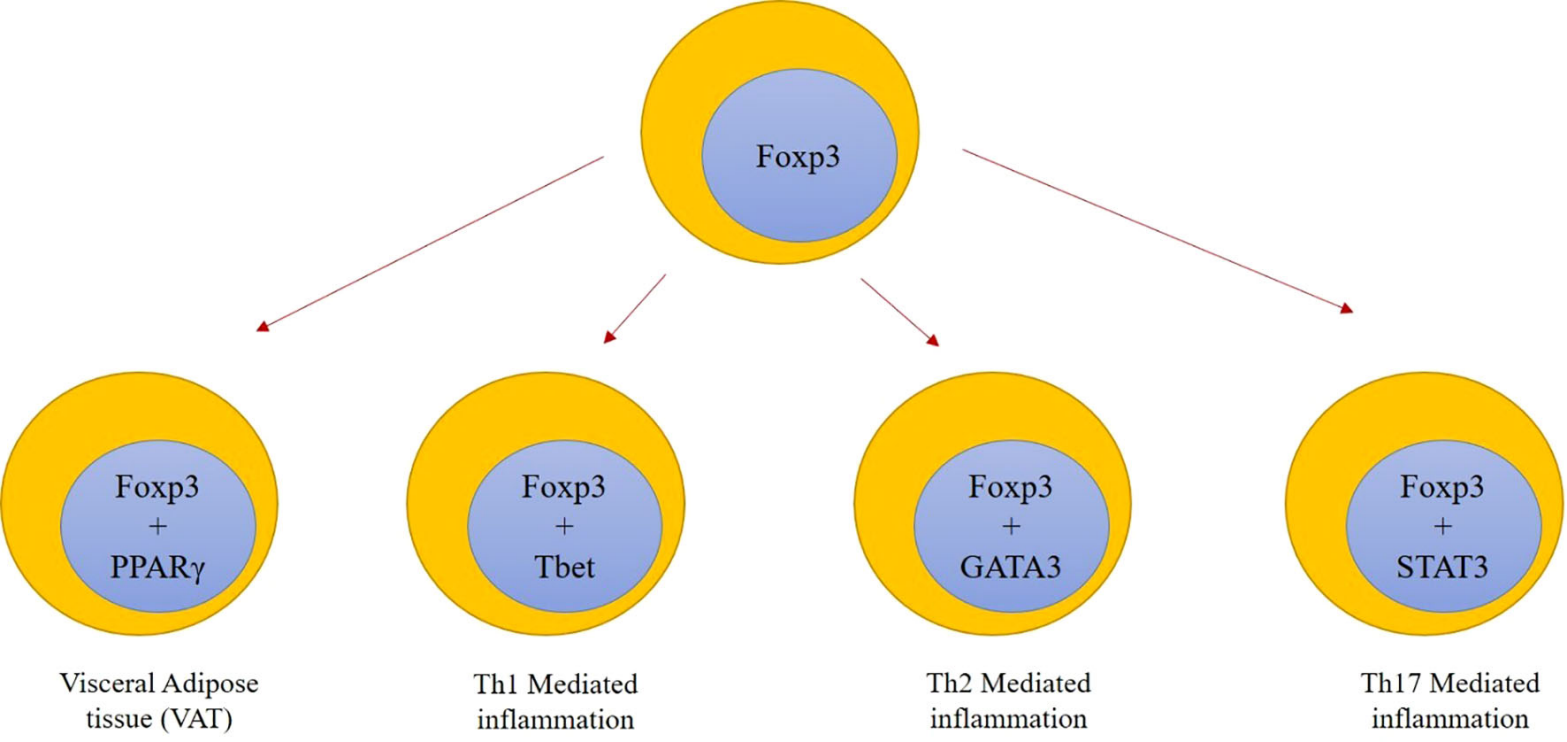
Depiction of inflammation control by specific nuclear factors. During low-grade inflammation, accumulation of FOXP3^+^PPARγ Treg cells in VAT further suppresses the other immune cell activity (macrophages and monocytes). VAT, visceral adipose tissue.

Visceral adipose tissue (VAT)—adipose tissue surrounding the organs—contains a higher number of T cells, B cells, macrophages (M2 phenotype), neutrophils, and Th2 cells, and a decreased number of Treg cells in those with diet-induced obese animal models (24–27). These results suggest that a distinct regulatory mechanism governing VAT immune cells exists that alters the functionality of Tregs. The production of IFN-γ from the dendritic cells of obese subjects has been shown to directly inhibit the VAT Treg production and insulin sensitivity (28). The location of fat depots in VAT has an impact on the development and origin of Tregs. In obese mouse models, VAT Tregs show increased expression of IL-33 receptor, ST2, indicating the IL-33/ST2 axis plays a mechanistic role. Thus, the location of VAT and VAT dendritic cells contribute in controlling the Treg population (24, 29). **Figure 2** depicts the control of inflammation by specific nuclear factors.

Adipose tissue Tregs regulate the balance between immunity and metabolism during diabetes. VAT Tregs cause insulin resistance and other features of metabolic syndrome, including dyslipidemia, endothelial dysfunction, and decreased nitric oxide generation, because of adipose tissue-derived pro-inflammatory cytokines, IL-6, and interferons (13, 30). VAT Tregs have also been shown to be implicated in the regulation of body weight, adipocyte hypertrophy, and glucose intolerance in diabetic mouse models (30, 31).

**Figure 3:**
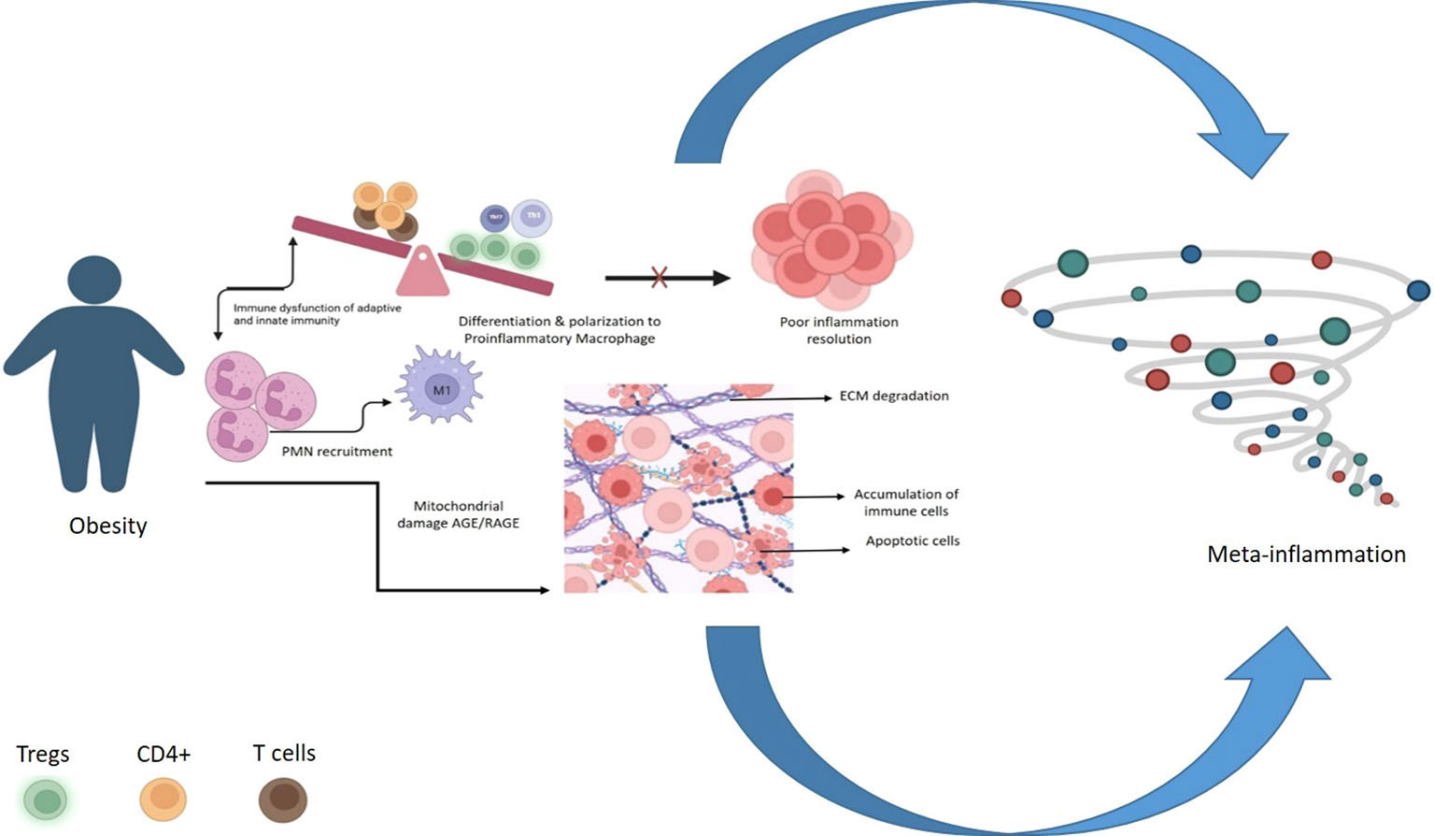
Effects of reduced Treg activity in diabetes: During diabetes dysfunction in adaptive & innate immunity and recruitment of neutrophils leads to poor resolution of inflammation which further leads to Metabolic inflammation (Meta-Inflammation).

In general, Tregs promote tissue repair and regeneration by their regulatory action on both innate and adaptive immune systems. Treg cells neutralize inflammatory cytokine secretion, promote phagocytosis of dead neutrophils, stimulate macrophage polarization toward an anti-inflammatory phenotype (M2) (32), and enhance wound healing and tissue regeneration (33). Based on these results, it can be inferred that impaired Treg function could be the reason for the delayed wound healing in diabetes. The balance between the Th1 and Th17 pro-inflammatory cells *vs*. Treg cells is important to control tissue damage caused by inflammatory responses. This is supported by the observation that with a significant reduction in the ratios of CD4^+^CD25^hi^, Treg/Th17, and CD4^+^CD25^hi^, Treg/Th1 is present in T2DM (34). However, this has been disputed in some studies. The observation that changes in the levels of effector memory T cells can aggravate the risk of cardiovascular complications during diabetes is in support of the observation that an imbalance between CD4^+^CD25^hi^, Treg/Th17, and CD4^+^CD25^hi^, Treg/Th1 occurs in these patients (35). Most studies have claimed that Treg levels were reduced in type 1 diabetes, whereas in type 2 diabetes of early duration (below 10 years), Treg levels showed no significant change. However, when the disease progresses, Treg levels were reported to decline in type 2 diabetes, including in conditions such as diabetes nephropathy (36, 37). Reduced levels of CD39^hi^Treg cells in obesity and T2DM change the various anti-inflammatory cytokines produced by the Tregs. IL-10 and TGF-β, which are needed for the proper functioning of Tregs, seem to play a critical role in the pathophysiology of diabetic complications like nephropathy and retinopathy. The differential expression of TGF-β during diabetes affects the expression of FOXP3 and IL-10, which, in turn, influence the proliferative activity of T cells, implying that Treg cell function is impaired in type 2 DM (38). **Figure 3** indicates that reduced Treg activity in diabetes could be one of the reasons for the persistent chronic low-grade inflammation.

## 4 Resolvins in obesity

SPM levels were found to be lower in adipose tissues of obese mouse models. RvD2 reduced local inflammation by controlling adipokine production and monocyte activity in these animal models. Enhanced inflammation resolution was reported in macrophages of patients with Diabetes mellitus related Acute ischemic stroke (DM–AIS) (acute ischemic stroke). The reason for the enhanced inflammation in the macrophages has been attributed to the differences in the levels or ratio of SPMs *vs*. pro-inflammatory molecules. RvD2/LTB4 levels were found to be significantly altered in AIS with DM, suggesting that RvD2 can reverse the effects of revascularization in diabetic mice and is involved in lowering adiposity and enhancing glucose tolerance. Despite these lines of evidence, the possible mechanisms for the inflammation resolution in these instances still remain elusive (39).

Dietary supplementation with PUFAs has shown to have some positive effects in diabetic patients. RvD1 promotes neuronal growth, helps prevent diabetic neuropathy by reducing inflammation, and slows glomerular dysfunction while lowering albuminuria during kidney issues. Fish oil (rich in EPA and DHA) may aid in enhancing the formation of resolvins and is likely to prevent endothelial damage in diabetes by inducing NO release. It has been suggested that NF-κB and NLRP3i serve as the downstream signaling processes in diabetic retinopathy that can be inhibited by RvD1 and, thus, may be of benefit in halting retinopathy (13, 40, 41). It is noteworthy that the supplementation of EPA and DHA showed few positive effects in enhancing the health status. This could be attributed to failure in the generation of adequate amounts of resolvins following EPA and DHA administration because of defects in the activities of COX and LOX enzymes and associated dyslipidemia in these instances.

**Figure 4:**
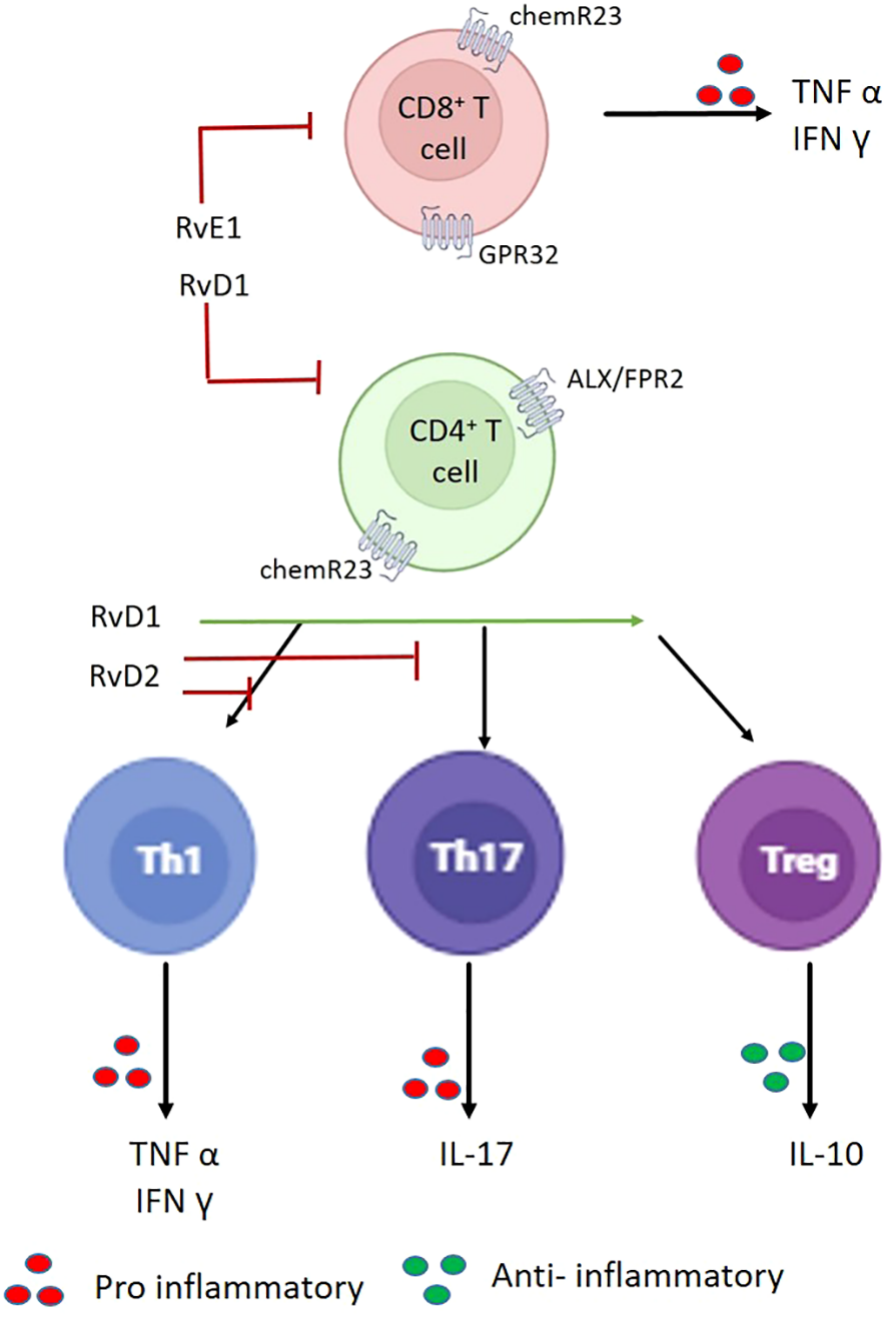
Resolvins influence on T cells: RvE1 prevents the infiltration of CD8^+^ and CD4^+^ T cells. D-series resolvins (RvD1) suppress the inflammatory responses of CD8^+^ T, T helper (Th)1, and Th17 cells; in addition, resolvins promote the conversion into T regulatory (Treg) cells.

During acute inflammation, dendritic cells (DCs) differentiate to secrete pro-inflammatory cytokines and PGE2. Dendritic cell differentiation in the presence of PGE2 induces preferential Th17 differentiation and activates naïve T cells. DCs exposed to RvE1, especially during differentiation, acquire the capacity to induce apoptosis of activated T cells through the induction of TLR and TGF-β signaling pathways (42, 43). RvD5 facilitates Treg differentiation by suppressing Th17 differentiation, promoting resolution of inflammation in rheumatoid arthritis, a model for chronic inflammation like diabetes (44). **Figure 4** shows the involvement of resolvins in deciding the T-cell fate from naïve CD cells.

Reduction in the levels of RvD1 is observed in a hyperglycemic state, which could be due to a defect in the production of key enzymes or in the interactions of receptors for resolvins. This could be overcome by the use of a compound 49b, which activates the beta-adrenergic receptor pathway. *In vitro* studies using retinal cells under hyperglycemic conditions have shown that compound 49b (a β-adrenergic receptor agonist, effective against diabetic retinopathy) effectively restored RvD1 function. This finding suggests that combinatorial therapy of RvD1 and beta-adrenergic receptors could be of significant benefit (45). Resolvin D1, Resolvin D2, and Maresin 1 modulate adaptive immune responses in human peripheral blood lymphocytes by reducing the pro-inflammatory cytokine production from activated CD8 T cells and CD4 T-helper (Th) 1 and Th17 cells by preventing the differentiation of naïve CD4 T cells to T helper cells. This results in the downregulation of T-bet and acts via GPR32 and ALX/FPR2 receptors, leading to the *de novo* generation of FOXP3^+^ Tregs. These findings indicate that the actions of specific SPMs on chronic inflammation and SPMS can directly modulate the inflammatory responses of already existing activated Th1 and Th17 cells (46). Similar increases in the percentage of Tregs were also observed in the mouse models of ischemia/reperfusion-induced acute kidney injury. The reason for the increase in Tregs could be attributed to the proliferation of pre-existing Tregs (13).

RvE1 is observed to be involved in the thermogenesis process, which improves the metabolic homeostasis (47), reduces pro-inflammatory markers in human pancreatic islets *in vitro* (48), and increases the expression of GLUT-4 (glucose transport), PPAR-γ (gene involved in insulin sensitivity), and insulin signaling pathways (49).

Ganglion cell apoptosis and increased vascular permeability are the hallmarks of diabetic retinopathy (DR). Exogenous injection of RvD2 minimized these conditions along with other inflammatory factors in streptozotocin-induced diabetic mice, suggesting that RvD2 protects the retina from the diabetic condition (50) and could be used as an alternative therapy. In a similar fashion, RvD1 is capable of reducing oxidative stress levels and inflammation by enhancing Brain-derived neurotrophic factor (BDNF) levels through the *PDX* gene (51).

RvD1 protects from retinal ageing, which in turn activates ALX/FRP2 receptor in photoreceptor cells, reduces high glucose-induced mitochondrial damage, decreases the levels of VEGF and proangiogenic miRNAs; RvD1 interacts with sirtuin 1, which has shown to be associated with various retinal pathologies. RvE1 and RvD1 reduced the expression of vascular cell adhesion molecule-1 (VCAM-1), IL-8, inflammatory macrophage protein-1 β (MIP-1), and TNF-α from Choroid-retinal endothelial cells (CRECs) or co-culture of CRECs and leukocytes (52). RvD1 is associated with controlling miRNA–exosome–cell proliferation in DR by modulating the expression of reactive oxygen species (ROS)-induced NF-κB signaling by FPR2 receptor, which leads to changes in the intracellular miRNAs in primary retinal photoreceptors, indicating that FPR2 receptor plays a crucial role in the action of RvD1 on VEGF (53). A difference in the levels of RvD1 was observed, which showed that males have a lesser amount compared to females, and the levels gradually decreased in the retina of both genders as their age progresses (54). TGF-β induced Tregs further help in the clearance of apoptotic T cells. Insulin resistance is decreased by RvD1 through the PI3–Akt–mTOR pathway, which acts by modulating Gsk-3b, an insulin signaling molecule. The changes in the neuronal hippocampus and hypothalamus during diabetes were also prevented by RvD1 (55). In high-fat diet (HFD)-induced mice, RvE3 increased the insulin signaling pathway and glucose uptake of the adipocytes in a dose-dependent manner. Although the exact mechanism is unknown, RvE3 is shown to enhance the phosphorylation of the AKT pathway, thereby increasing the glucose uptake and reducing the insulin resistance in adipocytes (56).

## 5 Molecular and cellular signaling mechanisms of Tregs and resolvins in physiological and disease states

**Figure 5:**
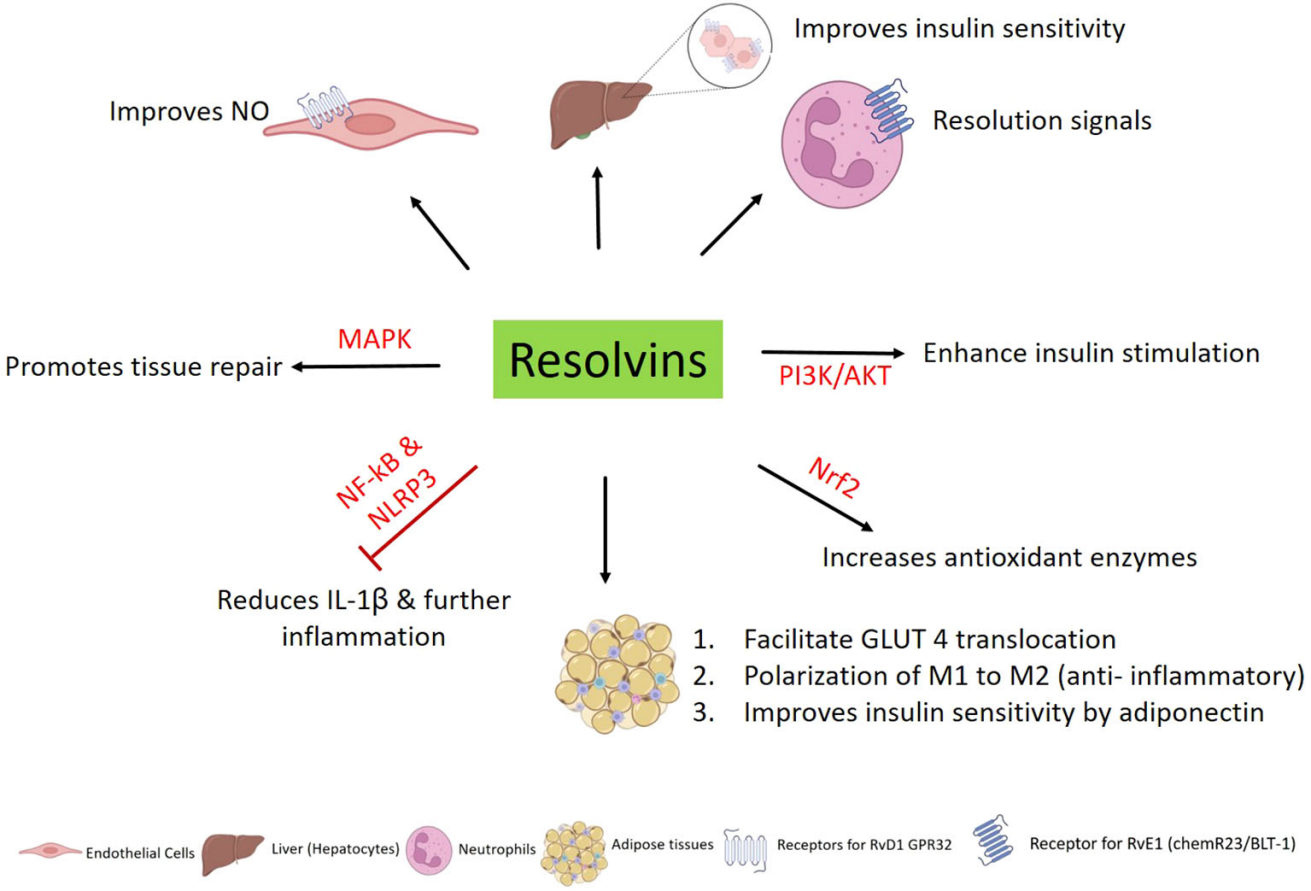
Downstream signaling pathways and cellular interactions of resolvins: Resolvins help in improving the insulin sensitivity in endothelial cells. RvD1 has been shown to improve NO, which is essential for insulin signaling and also combat oxidative stress. RvE1 boosts resolution signals via neutrophils. Resolvins act on PI3K/AKT, MAPK, and Nrf2 pathways, promoting tissue repair and insulin sensitivity, inhibiting inflammation by reducing the IL-1β secretion via NF-κB and NLRP3 inflammasome pathways.

Resolvins of D series mediate their actions through four key GPCRs: ALX/FPR2, GPR32, GPR18, and GPR37 (**Table 1**). Several studies have documented that resolvins of D series connect with cells through ALX/FPR2 receptors in mice, whereas they interact with the cells through GPR32 receptors in humans (13, 57). Experiments have shown that RvD2 signals through the GPR18 receptor (58), whereas RvD3 and RvD5 signal through GPR32 (59). GPR32 signaling in humans is atheroprotective, as it reduces neutrophil penetration and improves the clearance of dead cells inside the plaque (59). Resolvins of the E series interact through ChemR23 (also referred to as ERV1 or chemerin1), particularly in macrophages, neutrophils, and dendritic cells, and also act as a BLT1 receptor antagonist, which causes neutrophil apoptosis and decreases the effector T-cell recruitment (60, 61). Chiurchiù and colleagues reported that RvD1 and RVD2 increase the synthesis of FOXP3^+^ Tregs and thereby reduce the inflammation in CD4^+^ and CD8^+^ T cells following T-cell receptor stimulation and inhibit cytokine production by circulating Th1 and Th17 cells (46). Previous studies have shown that pro-inflammatory cytokines, especially TNF-α and IL-6, contribute to insulin resistance and lead to complications in type 2 diabetes (62–64). Further, SPMs primarily RvD1 have shown notable drug potential by signaling through the ALX/FPR2 receptor/GPR32 to alleviate inflammatory responses by inhibiting microglial activation in the central nervous system (CNS) (65). Accumulating evidence shows that SPMs support the anti-inflammatory process in macrophages and stabilize Tregs, contributing a protective machinery, and also serve as promising therapeutic molecules to suppress low-grade systemic inflammatory chronic disorders (62, 66, 67). It was shown that in type 2 diabetes, an imbalance in the immune profile with decreased FOXP3^+^ Treg cells along with over-activated Th1/Th17 cells occurs (68, 69). In addition, resolvins maintain the Th17/Treg balance through GPR32-mediated signaling (59). **Figure 5** provides an overview of the downstream signaling pathways and cellular interactions of resolvins.

**Table 1:** Metabolic effects of resolvins.

Types of resolvins	Receptors	Immune targets	Metabolic effects in diabetes	Model	Reference
D1	FPR2/ALX	Macrophages	Stimulate macrophage phagocytosis, enhancing faster wound healing and inflammation resolution	Diabetic mouse model	(76)
		Adipose tissue	Improves insulin sensitivity by decreasing the accumulation of pro-inflammatory type macrophages	Obese diabetic mouse model	(62)
	GPR32	Macrophages	Polarization of macrophages to resolution type and reduced pro-inflammatory cytokines	Human macrophages (*in vitro*)	(77, 78)
D2	GPR 18	Macrophages	Polarization of macrophages toward anti-inflammatory type	Mouse model	(79)
D3	GPR32	Unknown	Alleviates insulin resistance	*In vitro* and animal studies	(80)
D4	Unknown	Unknown	Unknown	Unknown	
D5	GPR101	Microglial and endothelial cells	Improves insulin resistance	Diabetic-induced obese mouse model	(81)
D6	Unknown	Unknown	Corneal nerve regeneration and wound healing	Animal model (rabbit)	(82)
E1	BLT-1	Unknown	Unknown	Unknown	
	ERV-1/ChemR23	Neutrophils and adipocytes	Enhanced cellular phagocytosis improves insulin sensitivity, glucose tolerance, and metabolic homeostasis, and reverses hyperinsulinemia and hyperglycemia	*In vitro* and animal studies	(47, 83, 84)
E2	BLT-1	Neutrophils	Suppress immune cell infiltration in adipose tissue	Animal studies	(85)
E3	BLT1R	Adipocytes	Improve insulin sensitivity and glucose homeostasis	Animal studies	(56)
E4	Unknown	Unknown	Unknown	Unknown	

It has been reported that a lower percentage of peripheral CD4^+^CD25^+^FOXP3^+^ Treg is commonly found in type 2 diabetes, mainly due to the reduced availability of resolvins (68). *In vivo* studies have revealed that RvD1 enhances the percentage of Tregs, improves renal tubular injury, and decreases the levels of TNF-α, IFN-γ, and IL-6 in the serum of the IRI-AKI mouse model (70). RvE1 signaling via ChemR23 inhibits the activation of NF-κB and cytokine secretion, such as TNF-α, IL-1β, and IL-6, which are known drivers of vascular dysfunction in diabetic patients (71).

**Figure 6:**
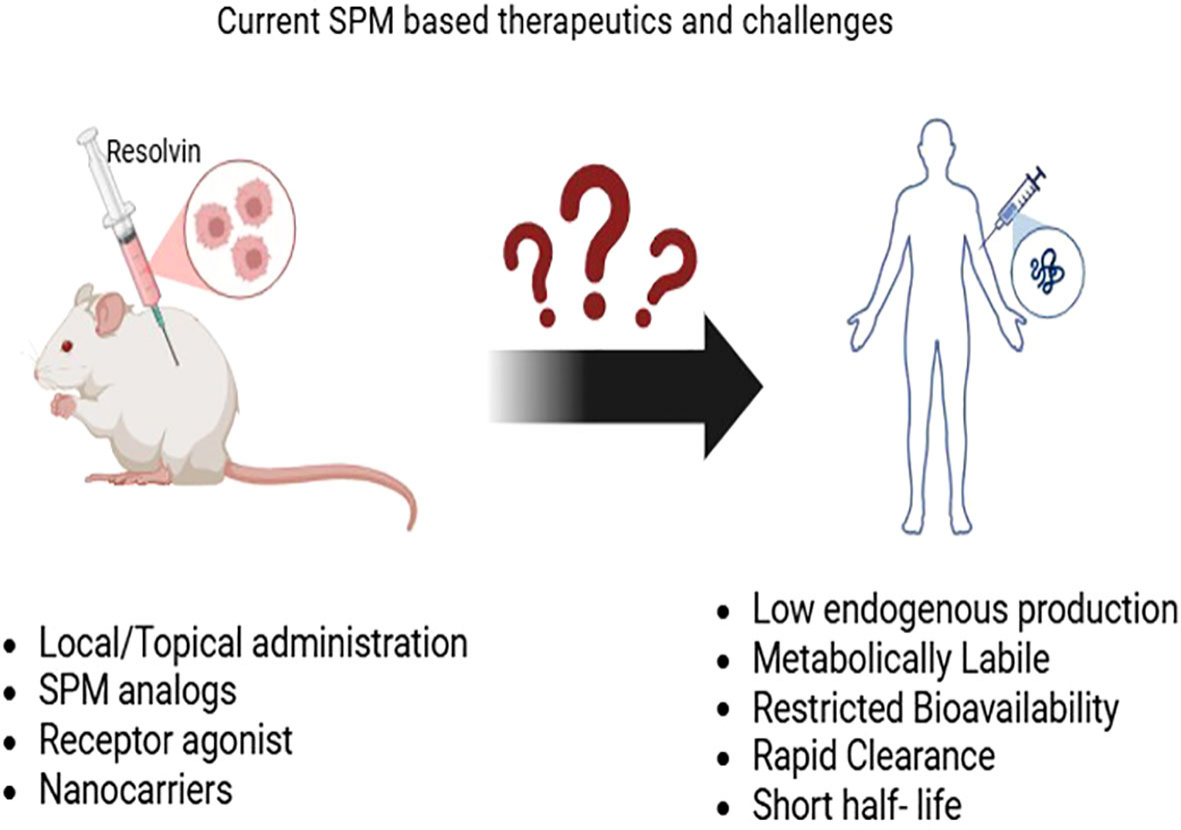
Current SPM-based therapeutics and human translational challenges. SPM, specialized pro-resolving mediator.

In obesity, immune responses mediated through the IL-10/STAT3 axis displays pivotal role for maintaining crosstalk between adipocytes and immune cells to regulate adipose tissue homeostasis (72), whereas Treg-secreted IL-10 is shown to facilitate obesity and insulin resistance via Blimp-1/IL-10 axis (73), presumed due to excessive activation of this cytokine in response to inflammation. However, RvD1 supplementation to inflamed obese VAT cells limited this excessive activation of the IL-10 by reducing the phosphorylation of STAT proteins and other target inflammatory genes of IL-10 without affecting its anti-inflammatory response (74), suggesting that RvD1 can tailor the quantitative and qualitative responses of human inflamed adipose tissue and provide a mechanistic basis for the immune-resolving actions. In spite of that, SPMs, including resolvins, can promote or inhibit T-cell differentiation, thereby directly regulating the T cell-mediated immune responses (75). Clinical studies have shown that cytokine-based interventions boost the Treg population in type 1 diabetes; however, their relevance to type 2 diabetes remains limited. Metainflammation, oxidative stress, and lifestyle-associated factors are the primary drivers in the progression from insulin resistance to type 2 diabetes. Moreover, available human studies are largely restricted to systemic blood-level observations, with less insight into the tissue or cellular level. Therefore, more translational studies in humans are essential (**Figure 6**) to investigate the therapeutic potential of Treg-resolvin interactions in preventing the onset of type 2 diabetes and their complications.

## 6 Conclusion and future perspective

Obesity and insulin resistance are associated with chronic inflammation, oxidative stress, and dysfunctional adipocytes. Various regulators like FOXP3 and IL-33 play key roles by impacting immune cell function in visceral adipose tissue and contributing to metabolic disorders. Dysfunctional Tregs in VAT are associated with the inflammatory state, impacting immune cell balance, insulin resistance, and metabolic syndrome. Treg cells play a vital role in tissue repair by regulating immune responses, affecting wound healing. Imbalances in Treg cell ratios and cytokine levels may contribute to delayed wound healing in diabetes. However, SPMS can directly modulate the inflammatory responses of existing and activated Th1 and Th17 cells. The relationship between chronic low-grade inflammation and diabetes is well known. In preclinical and *in vitro* models, RvD1 and RvE2 have been shown to improve insulin sensitivity and reduce meta-inflammation by partly increasing the VAT Tregs and FOXP3^+^ Tregs. However, little information is available in human studies and warrants further exploration. Various lines of evidence suggest that insulin resistance could be due to altered resolution of inflammation. Using the resolvin molecules and Tregs to target the inflammatory resolution phase could provide more insights. Future studies and translational to human use requires understanding the 1) target receptors and effector cells that becomes altered during resolution of inflammation, 2) clinical relevance of these molecules in treatment alone or in combination with various types of resolvin molecule, and 3) interactions and changes in these molecules that may affect resolution phase as diabetes progresses could provide deeper understanding and treatment options for insulin resistance and various diabetic complications.
